# The quality-of-life burden of knee osteoarthritis in New Zealand adults: A model-based evaluation

**DOI:** 10.1371/journal.pone.0185676

**Published:** 2017-10-24

**Authors:** J. Haxby Abbott, Ilana M. Usiskin, Ross Wilson, Paul Hansen, Elena Losina

**Affiliations:** 1 Centre for Musculoskeletal Outcomes Research, Department of Surgical Sciences, Dunedin School of Medicine, University of Otago, Dunedin, New Zealand; 2 Orthopaedic and Arthritis Center for Outcomes Research, Department of Orthopaedic Surgery, Brigham and Women’s Hospital, Boston, MA, United States of America; 3 Department of Economics, University of Otago, Dunedin, New Zealand; University of Umeå, SWEDEN

## Abstract

**Background:**

Knee osteoarthritis is a leading global cause of health-related quality of life loss. The aim of this project was to quantify health losses arising from knee osteoarthritis in New Zealand (NZ) in terms of quality-adjusted life years (QALYs) lost.

**Methods:**

The Osteoarthritis Policy Model (OAPol), a validated Monte Carlo computer simulation model, was used to estimate QALYs lost due to knee osteoarthritis in the NZ adult population aged 40–84 over their lifetimes from the base year of 2006 until death. Data were from the NZ Health Survey, NZ Burden of Diseases, NZ Census, and relevant literature. QALYs were derived from NZ EQ-5D value set 2. Sensitivity to health state valuation, disease and pain prevalence were assessed in secondary analyses.

**Results:**

Based on NZ EQ-5D health state valuations, mean health losses due to knee osteoarthritis over people’s lifetimes in NZ are 3.44 QALYs per person, corresponding to 467,240 QALYs across the adult population. Average estimated per person QALY losses are higher for non-Māori females (3.55) than Māori females (3.38), and higher for non-Māori males (3.34) than Māori males (2.60). The proportion of QALYs lost out of the total quality-adjusted life expectancy for those without knee osteoarthritis is similar across all subgroups, ranging from 20 to 23 percent.

**Conclusions:**

At both the individual and population levels, knee osteoarthritis is responsible for large lifetime QALY losses. QALY losses are higher for females than males due to greater prevalence of knee osteoarthritis and higher life expectancy, and lower for Māori than non-Māori due to lower life expectancy. Large health gains are potentially realisable from public health and policy measures aimed at decreasing incidence, progression, pain, and disability of osteoarthritis.

## Introduction

Osteoarthritis (OA) is a highly prevalent condition that can result in disabling pain and loss of physical function. New Zealand (NZ) data confirm that physician-diagnosed and symptomatic OA increases with age, from at least 8% of people aged 45–55 years to 30% aged over 75.[1] The most common site of OA is the knee;[2] knee OA is a leading cause of global disability and is associated with significant economic costs as well as reduced quality of life.[3, 4] United States (US) data indicate that the incidence of knee OA peaks in the 55–65 age group at around 0.4% annually and has an average incidence of approximately 0.25% from age 25 to 85 years.[5] These rates correspond to an annual incidence of approximately 7,000 people in the 2013 NZ resident population (totaling approximately 2.8 million people aged 25 year and over).

There have been few studies quantifying the health losses due to OA in the NZ population, and none have looked specifically at the burden of knee OA. The NZ Burden of Diseases, Injuries and Risk Factors Study (NZBD) reported that all-site OA was responsible for the loss of 20,738 disability-adjusted life years (DALYs) in 2006,[6] calculated using standardized disability weights[7] for moderate and severe OA from the Global Burden of Disease (GBD) study.[4] However, quality-adjusted life years (QALYs)–which use a range of available health related quality of life (HRQoL) measurements–are more commonly used for economic evaluations.[8] Several descriptive systems for HRQoL are available, of which the EQ-5D[9] is by far the most widely used internationally.[10] The EQ-5D and the value set of Devlin et al.[11] are widely used for estimating QALYs in NZ, and are recommended by the Pharmaceutical Management Agency (PHARMAC) as the preferred metric for measuring health-related quality of life and for conducting economic evaluations in NZ.[8]

This paper reports on a study to quantify health losses arising from knee OA in NZ in terms of QALYs lost. Consistent with the above-mentioned international literature, differences in these losses are tested for age and sex effects. In addition, the effect of ethnic group–Māori (NZ’s indigenous minority) versus non-Māori–is investigated; ethnicity is a potentially important factor because Māori people are well known to suffer higher health losses for most health conditions than non-Māori people.[6]

## Materials and methods

### The OAPol model

The Osteoarthritis Policy Model (OAPol), a validated Monte Carlo computer simulation model of the natural history and management of knee OA,[5, 12–14] was used to estimate the QALYs lost due to knee OA in the NZ adult population over their lifetimes. This model generates simulated cohorts of individuals based on user-defined inputs for age, sex, and knee OA severity and pain. Each simulated individual passes through a sequence of annual transitions that model the clinical course of knee OA from one health state to another, without interventions, corresponding to HRQoL improvements or deteriorations. The HRQoL state transitions are based on the Western Ontario and McMaster Osteoarthritis Index (WOMAC) pain score (see Pain and health-related quality of life, below). Details about the health-state transitions in the OAPol model have been published elsewhere.[14, 15]

### Cohort characteristics

The data used to define our simulated cohort were sourced from the NZ Health Survey, NZBD, Injuries and Risk Factors Study, NZ Census, and other relevant literature (see S1 Appendix for details about data sources).[6, 16–18]

The NZ adult population aged 40–84 was modelled over their lifetimes from the base year of 2006 to death in two cohorts: one with knee OA and another without knee OA. Each cohort was sub-divided into 5-year age ranges (e.g. 40–44, 45–49, etc), and the mean BMI for each sex and ethnicity group (Māori or non-Māori) was derived from 2006 NZ Health Survey (NZHS) data.[18] Mortality data for each age, sex, and ethnicity group were compiled from the NZ life tables that provide the probability that a person will die within 5 years.[16]

### Pain and health-related quality of life

In the OAPol model, knee pain is represented by the WOMAC pain score, which is a five-item questionnaire scored in the range 0–100, where 100 is the highest level of pain.[19] Subjects with knee OA were initialised with a mean WOMAC pain score of 30 (standard deviation 15), and an increase of 0.5 points (standard deviation 5) for each subsequent year. The mean WOMAC pain level of 30, based on cohort research data,[20, 21] was used to generate a cohort with mild to moderate pain from knee OA with substantial variation around the mean. The subsequent year increase in pain was used to give subjects a relatively stable pain trajectory over the course of their OA progression.[21] Based on the WOMAC score, individuals were classified into three levels of pain severity—no pain (WOMAC < 1), moderate pain (1–70), and severe pain (>70)—each with an associated (age-specific) HRQoL value in the range 0–1, where dead = 0 and perfect health = 1.

To determine HRQoL values for each level of pain, we proceeded in two steps. First, using data collected in a 1999 survey of the NZ adult population that asked respondents to rate their own current health on the five EQ-5D dimensions,[22] we stratified all responses by 5-year age range and by the level of pain reported. For each of these sub-groups, we then calculated the mean HRQoL value using two HRQoL valuations: the NZ EQ-5D value set 2, derived from visual analog scale (VAS) valuations of EQ-5D health states, as recommended by PHARMAC for health technology assessments;[23, 24] and, a transformation of these values designed to more closely approximate valuations elicited using trade-off methods such as the standard gamble (SG) as there have been reports suggesting that VAS scores may be more responsive to QALY loss relative to scores elicited using SG methodology.[25] We applied both the untransformed and the transformed HRQoL values to provide alternative estimates for the health loss associated with knee OA pain, and to aid comparisons across other literature. The derived HRQoL values for each age group and level of pain are reported in Table 1, and Table B in S1 Appendix, for the untransformed and transformed values, respectively.

**Table 1:** Quality of life utilities, by age and pain level.

	Pain level		
Age	No pain or discomfort	Moderate pain or discomfort	Extreme pain or discomfort
**40–44**	0.959	0.652	0.314
**45–49**	0.959	0.642	0.285
**50–54**	0.951	0.646	0.239
**55–59**	0.946	0.649	0.122
**60–64**	0.968	0.643	0.160
**65–69**	0.947	0.627	0.280
**70–74**	0.974	0.623	0.239
**75–79**	0.950	0.635	0.165
80–84	0.916	0.609	0.107

Note: QALY input was taken from the EQ-5D Tariff 2 health state preference values (Devlin, Hansen et al. 2003) derived from a survey of the NZ population (Devlin, Hansen et al. 2000). We stratified the original EQ-5D data set by level of pain (no pain or discomfort; moderate pain or discomfort; extreme pain or discomfort).

For cohorts without knee OA, HRQoL values were determined based on the population distribution of pain not due to OA. In the absence of published NZ estimates, the proportion of the total population in pain was estimated using general population data from the North Staffordshire Osteoarthritis Project (NorStOP).[26] Based on the NorStOP findings, we applied the assumption that 90% of the population in pain not due to OA are experiencing moderate pain and 10% severe pain. The detailed derivation for the distribution of pain for people without knee OA is described in the Technical Appendix (S1 Appendix).

### Knee OA prevalence

The prevalence of all-site OA (Table 2) was derived from the NZBD study, a partner to the GBD study.[1, 4, 6, 27] Data from the 2012 United States (US) National Health Interview Survey (NHIS) were used to determine the proportion of people with knee OA as a proportion of the number of people with OA at any site.[28] Details about the derivation of the prevalence of knee OA in the NZ population can be found in the Technical Appendix (S1 Appendix).

**Table 2:** Estimates of prevalence of knee osteoarthritis in New Zealand.

Ethnicity	Sex	Age Range								
		40–44	45–49	50–54	55–59	60–64	65–69	70–74	75–79	80–84
Non-Maori	**Male**	1.19%	4.82%	4.79%	6.27%	6.30%	11.40%	11.44%	12.75%	12.85%
	**Female**	0.80%	4.47%	4.47%	10.45%	10.41%	16.98%	16.92%	21.55%	21.50%
Maori	**Male**	0.86%	2.88%	2.88%	9.72%	9.72%	14.43%	14.43%	20.84%	20.84%
	**Female**	1.29%	4.83%	4.83%	6.98%	6.98%	13.10%	13.10%	18.34%	18.34%

Note: Prevalence of OA was taken from the New Zealand Burden of Diseases, Injuries and Risk Factors Study (NZBD) study (Ministry of Health 2013), a partner to the GBD study (Murray, Vos et al. 2012, Cross, Smith et al. 2014). We used data from the 2012 United States (US) National Health Interview Survey (NHIS) to determine the proportion of people with knee OA as a proportion of the number of people with OA at any site (United States National Health Interview Survey 2012) and adjusted downward to account for self-report bias (March et al. 1998) (see S1 Appendix).

### QALYs lost

QALYs lived for each individual were calculated as the sum of HRQoL values for each year of life. For people with and without knee OA, quality-adjusted life-expectancy (QALE) was estimated, stratified by age (5-year bands), sex, and ethnicity (non-Māori, Māori). For each of these sub-populations, the QALYs lost due to knee OA were calculated by subtracting the QALE of people with knee OA, weighted by the proportion of people in the group with prevalent knee OA, from the QALE of the same sub-population without knee OA.

### Sensitivity analyses

A key driver of modeled QALY losses attributable to knee OA is the difference in pain between people with and without knee OA. Therefore probabilistic sensitivity analyses (PSA) were performed to understand the impact of the assumption that 90% of the people in pain not due to knee OA are in moderate instead of extreme pain. PSA was conducted using a uniform distribution from 80% to 100% moderate pain for those in pain not due to knee OA.

Sensitivity analysis was also performed by varying knee OA prevalence values. The prevalence estimates reported by the NZBD study are based on self-reports of the presence of physician-diagnosed OA; however there is some evidence that self-reported OA may be biased upward.[29] March et al. (1998) report the false-positive rate of self-reported OA to be 0–19.3%.[29] For the base case, we used prevalence rates conservatively adjusted downward using the positive predictive values (PPVs) from the study by March et al.[29] We checked the impact of this assumption by conducting PSA (as above) by also running the model with unadjusted prevalence values as reported in the NZBD study.

## Results

### Primary analysis

For people in the NZ population aged 40–84 without knee OA, their average life expectancy is 20.67 QALYs, weighted by the proportion of the NZ population in each age-, sex-, and ethnic group. The weighted average life expectancy for the NZ population aged 40–84 with knee OA is 12.14 QALYs. For the sub-population with knee OA (accounting for age, sex, ethnicity, and non-OA-related pain), the predicted QALE if they did not have knee OA is 15.57 QALYs. This corresponds to a difference of 3.44 QALYs, which represents the QALY loss per person due to knee OA (Table 3). This individual-level estimate corresponds to a total of 467,240 QALYs lost across the adult population from 2006 until death (Table 3).

**Table 3:** Summary of per-person and population-based QALY losses due to knee OA in New Zealand.

	Weighted QALE in people with no knee OA	Weighted QALE in people with knee OA	Weighted QALE if people with OA didn't have knee OA*	QALY loss per person with knee OA	Population-based QALY losses
Non-Maori Male	19.73	12.20	15.54	3.34	166,023
Non-Maori Female	21.96	12.22	15.77	3.55	272,568
Maori Male	17.57	10.05	12.64	2.60	11,562
Maori Female	19.78	12.17	15.55	3.38	17,087
**Total Population**	**20.67**	**12.14**	**15.57**	**3.44**	**467,240**

QALE = quality-adjusted life expectancy; QALY = quality-adjusted life year; OA = osteoarthritis

*The expected QALE if persons with knee OA didn’t have knee OA was used to determine the per-person QALY losses. It was calculated by weighting the QALE of persons without knee OA according to the age distribution of people with knee OA.

QALYs lost due to knee OA differ by sex, ethnicity, and age. QALY losses are higher for females than males, and lower for Māori than non-Māori (Table 4). Māori have lower QALY losses per person than non-Māori because of their lower average life expectancy. Overall QALYs lost due to knee OA are inversely related to people’s age: they are higher in younger age groups as a result of their longer life expectancy (Table 4). The proportion of QALYs lost out of the total QALE for those without knee OA is similar across all subgroups, ranging from 20 to 23 percent.

**Table 4:** Ethnicity, sex, and age-specific per-person and population-based QALY losses due to OA, assuming 90% moderate pain for subjects in pain not due to OA.

Ethnicity	Sex	Age	Proportion with knee OA	QALE in persons without knee OA	QALE in persons with knee OA	Per-person QALY losses in persons with knee OA*	Population-based QALE losses in persons with knee OA
Non-Maori	M	40–44	0.012	28.82	22.34	6.48	10551.97
		45–49	0.048	25.39	19.84	5.55	35422.91
		50–54	0.048	22.02	17.29	4.73	26514.94
		55–59	0.063	18.77	14.74	4.03	27745.57
		60–64	0.063	15.63	12.31	3.32	17814.60
		65–69	0.114	12.69	9.99	2.70	21586.74
		70–74	0.114	9.90	7.81	2.09	12914.12
		75–79	0.127	7.41	5.85	1.57	9131.25
		80–84	0.128	5.39	4.24	1.15	4341.30
		Total		15.54	12.20	3.34	166023.41
	F	40–44	0.008	31.33	24.03	7.30	8426.69
		45–49	0.045	27.92	21.52	6.40	39248.26
		50–54	0.045	24.53	18.96	5.57	29815.42
		55–59	0.105	21.19	16.37	4.82	56407.82
		60–64	0.104	17.92	13.85	4.06	36980.56
		65–69	0.170	14.73	11.43	3.30	41482.76
		70–74	0.169	11.61	9.06	2.55	25454.77
		75–79	0.216	8.75	6.84	1.92	22157.55
		80–84	0.215	6.32	4.95	1.37	12593.97
		Total		15.77	12.22	3.55	272567.81
Maori	M	40–44	0.009	23.41	18.49	4.93	814.83
		45–49	0.029	20.28	16.12	4.15	2002.33
		50–54	0.029	17.27	13.76	3.51	1303.93
		55–59	0.097	14.47	11.52	2.95	2890.25
		60–64	0.097	11.97	9.52	2.45	1610.46
		65–69	0.144	9.76	7.74	2.02	1575.02
		70–74	0.144	7.68	6.10	1.58	760.76
		75–79	0.208	5.82	4.60	1.22	455.82
		80–84	0.208	4.33	3.40	0.93	148.57
		Total		12.64	10.05	2.60	11561.96
	F	40–44	0.013	26.14	20.40	5.74	1578.25
		45–49	0.048	22.86	17.92	4.94	4433.65
		50–54	0.048	19.72	15.47	4.24	2858.42
		55–59	0.070	16.75	13.10	3.65	2767.53
		60–64	0.070	13.98	10.91	3.07	1577.79
		65–69	0.131	11.50	8.97	2.53	1965.30
		70–74	0.131	9.16	7.18	1.98	984.28
		75–79	0.183	7.04	5.51	1.53	669.54
		80–84	0.183	5.22	4.10	1.12	252.13
		Total		15.55	12.17	3.38	17086.88
**Totals:**				**15.57**	**12.14**	**3.44**	**467,240**

QALE = quality-adjusted life expectancy; QALY = quality-adjusted life year; OA = osteoarthritis

*The expected QALE if persons with knee OA didn’t have knee OA was used to determine the per-person QALY losses. It was calculated by weighting the QALE of persons without knee OA according to the age distribution of people with knee OA.

### Secondary HRQoL analysis

Using a Torrance transformation to adjust the HRQoL values results in lower QALY losses due to knee OA in the NZ population: on average, 1.65 QALYs per person (9% of the total QALE for those without knee OA) and 224,364 QALYs at the population level. Proportional losses were unchanged: as before, Māori and males have lower QALY losses than non-Māori and females; QALY losses per person are highest for non-Māori females, with a mean loss of 1.73 QALYs per person (Figure A in S1 Appendix).

### Sensitivity analysis

Sensitivity analyses with respect to the assumption that 90% of the people in pain not due to knee OA are in moderate instead of extreme pain were performed on the Torrance-transformed HRQoL values. The PSA using a uniform distribution from 80% to 100% moderate pain for those in pain not due to knee OA showed a 95% uncertainty interval (UI) of 1.42 to 1.86 QALYs lost per person. At the sub-population level, 95% UIs were from 1.34 to 1.77 QALYs for non-Māori males, 1.51 to 1.95 QALYs for non-Māori females, 0.91 to 1.85 QALYs for Māori males, and 1.34 to 1.85 QALYs for Māori females.

Using the unadjusted prevalence rates from the NZBD study, the mean loss due to OA is increased slightly from the above, to a UI of 1.52 to 1.94 QALYs per person.

## Discussion

At both the individual and population levels, knee OA is responsible for large QALY losses over people’s lifetimes in NZ. On average, knee OA accounts for 3.44 QALYs lost per person, corresponding to 467,240 QALYs across the adult population, based on NZ EQ-5D health state valuations. Due to both the high prevalence and the disablement attributable to this condition, large health gains are potentially realisable from public health and policy measures aimed at decreasing the incidence, progression, pain, and disability of knee OA.

The QALY losses due to knee OA arising from the NZ EQ-5D value set 2, the recommended value set for NZ for economic evaluations,[23] differ from some overseas estimates due to methodological differences resulting in different HRQoL values across countries. For example, Losina et al.[13] estimated mean losses of 1.71 QALYs per person in the US (approximately half the present study’s estimate of 3.44 QALYs per person). The alternative valuation in the present study, using Torrance transformed[25] HRQoL values to approximate SG-derived health status scores in contrast to VAS derived scores, produced QALY losses more closely aligned with those reported in the US,[13] and with DALY burden reported in the NZBD and GBD studies.[4, 30] Projecting the reported 2006 DALY burden of all-site OA to estimate lifetime DALY losses, using sex-, ethnicity-, and age-specific life expectancies from NZ population life tables, [16] gives a total lifetime burden of all-site OA of 377,000 DALYs; the Torrance-transformed QALY losses estimated by the OAPol model (224,364, Figure A in S1 Appendix) represent around 60 percent of this total (226,200), consistent with the ratio of knee to all-site OA used in this study. This contrast highlights the influence of health status valuations on disease burden estimates.

Strengths of this study include the use of an established, validated computer simulation model[5, 12, 13] populated with robust data sourced from Statistics New Zealand, including the NZBD study[1] (see S1 Appendix), and health status valuations based on Devlin et al.’s EQ-5D value set,[24] as widely used for economic evaluations in NZ.[23, 24] In contrast, the NZBD, Injuries and Risk Factors Study (NZBD) used only two levels of disability weights derived from international research, and estimated the annual cross-sectional DALY loss for a single year (2006).

Limitations of this study include the absence of knee-specific NZ OA prevalence data or knee OA-related pain. Knee OA prevalence was estimated from NZHS self-reported data on all-site OA by using the ratio of knee OA to all OA found in the US population, assuming the proportion of knee OA would be applicable to NZ. Prevalence estimates of OA can differ markedly depending on case definition.[31] The estimates utilised in the model–as reported in the GBD study–may have resulted in conservative estimate of QALE loss due to OA because, as noted by the GBD authors, these “may be a substantial underestimate of the true prevalence” (p. 1328 column 1),[4] and thus may underestimate the burden of disease for the purposes of public health projections and healthcare needs assessments.[32] The non-NZ sourced data on the population distribution of non-OA-related pain adopted provide relatively high estimates of the proportion of the general population experiencing moderate and severe pain, compared with unpublished NZHS data, and thus provide a conservative estimate of HRQoL decrement attributable to OA.

By 2041, the number of people over the age of 65 will double: from 650,000 (2014) to 1.28–1.37 million (2041), with the proportion of people over 65 having increased to 25% of the population.[33] Due to the confluence of the ageing population and rising rates of obesity, injury, sedentary lifestyles, and other factors, it is predicted that the number of people affected by OA will increase by around 50% over the coming two decades.[34, 35] Total joint replacements are among the most common elective surgical procedures and rates are increasing rapidly primarily due to the ageing population.[36] By 2026 total knee joint replacement surgeries in NZ are predicted to rise by 184%, on top of the 52% rise already evident between 2001 and 2010.[36] These data indicate that public health and policy measures aimed at decreasing the incidence, progression, pain, and disability of knee OA have the potential to produce large health gains.

### Conclusions

At both the individual and population levels, knee OA is responsible for large lifetime QALY losses. Based on NZ EQ-5D health state valuations, mean health losses due to knee OA over people’s lifetimes in NZ are 3.44 QALYs per person. Due primarily to life expectancy differences, QALY losses are higher for non-Māori females (3.55) than Māori females (3.38), and higher for non-Māori males (3.34) than Māori males (2.60). Overall, this corresponds to 467,240 QALYs lost due to knee OA over the lifetimes of the 2006 NZ adult population. Large health gains are potentially realisable from public health and policy measures aimed at decreasing incidence, progression, pain, and disability of OA.

## Supporting information

S1 Appendix (PDF)

## Abbreviations

DALY: disability-adjusted life year
EQ-5D: EuroQOL 5-dimension health-related quality of life instrument
GBD: Global Burden of Diseases study
HRQoL: health-related quality of life
NHIS: United States National Health Interview Survey
NorStOP: North Staffordshire Osteoarthritis Project
NZ: New Zealand
NZBD: New Zealand Burden of Diseases, Injuries and Risk Factors study
NZHS: New Zealand Health Survey
OA: osteoarthritis
OAPol: Osteoarthritis Policy model, a a validated Monte Carlo computer simulation model of the natural history and management of knee OA
PHARMAC: Pharmaceutical Management Agency
PSA: probabilistic sensitivity analyses
QALE: quality-adjusted life expectancy
QALY: quality-adjusted life year
SG: standard gamble
UI: uncertainty interval
US: United States
VAS: visual analogue scale
WOMAC: Western Ontario and McMaster Universities osteoarthritis index
