# Supplementary material for: The quality-of-life burden of knee osteoarthritis in New Zealand adults: A model-based evaluation
Source: PLoS One. 2017 Oct 24;12(10):e0185676. doi: 10.1371/journal.pone.0185676 (PMC5655469; doi:10.1371/journal.pone.0185676)
Supplement: S1 Appendix — (PDF) [file pone.0185676.s001.pdf]

The Burden of Knee Osteoarthritis in New Zealand: A Model-Based Evaluation

TECHNICAL APPENDIX

Part I: Data sources and derivation of input parameters

A. Data sources

New Zealand Census 2006: We used data from the New Zealand Census 2006 (http://www.stats.govt.nz/Census/2006CensusHomePage.aspx) for the usually-resident population of New Zealand, including breakdowns by age, sex, and ethnicity.[1] The census usually-resident population count of New Zealand is all people counted in New Zealand on census night, excluding overseas visitors and New Zealand residents temporarily overseas.

New Zealand Life Tables 2005-07: Every five years, Statistics New Zealand produces complete period life tables, using average mortality rates for three successive years centred on a census year. We used the 2005-7 period life tables for the Māori, non-Māori and total populations, which are centred on the 2006 New Zealand Census (http://www.stats.govt.nz/browse_for_stats/health/life_expectancy/new-zealand-life-tables-2005-07.aspx).[2]

New Zealand Health Survey: The NZHS is conducted by the New Zealand Ministry of Health.[3] It is designed to be a nationally-representative sample of New Zealanders. The survey used a multi-stage, stratified design to achieve a sample probability proportionate to the underlying population, with increased sampling of some ethnic groups. Interviewers began at a random point in each small, randomly selected geographic area (meshblocks). Interviewers selected every *k*th house for enrolment of one adult aged 15 years and over, and conducted the interviews in the participants' homes, at a time to suit participants.

Height, weight, and waist measurements were taken directly using weighing scales, stadiometer, and anthropometric measuring tape.

The New Zealand Burden of Diseases, Injuries and Risk Factors Study (NZBD) 2006–2016: The epidemiological data for the *NZBD* were derived from multiple sources: disease registers, linked or unlinked administrative databases, population-based health surveys including the *New Zealand Health Survey*, and epidemiological research studies.[4] *NZBD* epidemiologists filled gaps in the empirical data using DISMOD, a multi-state life-table software program.[5] In some cases, regression or other smoothing methods were applied to derive the required subpopulation estimates for diseases for which the available data were either prior to or later than 2006. The *NZBD* was used as the primary source for the prevalence of OA (personal communication, Michelle Liu, NZ Ministry of Health).

United States (US) National Health Interview Survey (NHIS) 2012. The NHIS is a cross-sectional household interview survey that covers the civilian non-institutionalized population residing in the US at the time of the interview. The NHIS is one of the major data collection programs of the National Center for Health Statistics (NCHS), which is part of the Centers for Disease Control and Prevention (CDC).[6] The main objective of the NHIS is to monitor the health of the US population through the collection and analysis of data on a broad range of health topics. A major strength of this survey lies in the ability to display these health characteristics by many demographic and socioeconomic characteristics. (source: http://www.cdc.gov/nchs/nhis/about_nhis.htm)

B. Prevalence of knee OA in NZ

We first obtained the prevalence of OA in NZ for all body sites using data from the New Zealand Burden of Disease Study, a partner to the Global Burden of Disease study[5] [7, 8].

The prevalence of OA from this study was stratified by age, sex, and race (Maori or non-Maori). Since this data gave the prevalence of OA at all sites (including hip, hand, and knee), we used data from the US NHIS 2012 to approximate the proportion of cases of all-site OA that occur in the knee, stratified by age and sex. For each age and sex category, we calculated a ratio of knee OA to all OA, and then weighted the age-and sex- specific ratios by the NZ population distribution by age to generate more stable estimates for sex-specific ratios.

Because there is evidence indicating that self-reported OA prevalence data overstates the true prevalence of diagnosed knee OA,[9] the NZBD-sourced prevalence estimates were deflated using published data on the positive predictive value (PPV) of self-diagnosis of symptomatic knee OA. We use PPV values generated from a study by March and colleagues, who reported a PPV of 78% for self-reported knee OA from a random sample of people in Sydney, Australia.[9] We used, as the primary input data, adjusted estimates for the prevalence of knee OA for each sex- age- and race-specific group calculated by multiplying the self-reported prevalence of OA by the sex-specific ratio of knee OA to all-site OA, and then multiplying by the PPV from March et al. A sensitivity analysis was also performed that used the unadjusted knee OA prevalence values (i.e. without using PPVs to adjust for self-reporting biases). It is assumed that between these two analyses would lie the prevalence estimates resulting from adjustment of the NZBD-derived estimates using both sensitivity and specificity of self-reported OA, or from utilizing negative predictive value (NPV) of self-reported OA; however sound estimates of these data are currently unavailable. Thus these two analyses serve as reasonable and conservative bounds for estimated knee OA prevalence.

C. HRQoL values for the New Zealand population

The HRQoL values were obtained from the NZ EQ-5D value set 2, as recommended by PHARMAC for economic evaluations.[10, 11] That value set was derived from a population-based survey in which respondents provided health-state preference valuations relating to a selection of 33 hypothetical health states described using the EQ-5D health state classification system.[12]

NZ HRQoL values were stratified to 3 pain levels corresponding to responses to the pain item within the EQ-5D. These strata were informed by data collected in a 1999 survey of the NZ adult population that asked New Zealanders to rate their own current health on the five EQ-5D dimensions.[13] The individual-level data were obtained from the original investigators,[13] and were stratified by 5-year age range and by pain level according to the three levels on the EQ-5D-3L instrument: (1) no pain or discomfort, (2) moderate pain or discomfort, and (3) extreme pain or discomfort. The resulting HRQoL values are reported in Table 2 of the manuscript text.

D. HRQoL values for people without knee OA

For cohorts without the presence of knee OA, we wanted the HRQoL values to reflect the fact that a certain proportion of the population will be in pain, even if not from knee OA. In order to account for this, we weighted the HRQoL values by the proportion of the general population that is in pain due to causes other than knee OA.

The proportion of the NZ population that is in pain due to reasons other than OA was estimated using published data from the North Staffordshire Osteoarthritis Project (NorStOP). NorStOP provides estimates of the proportion of adults over age 50 who report being in pain in the last four weeks, stratified by 10 year age ranges and by sex.[14] We assumed that those aged 40-50 years would have similar levels of pain to those aged 50-55

years. To estimate the proportion of people in pain not due to knee OA, we subtracted the proportion of people with symptomatic knee OA (Table 2) from the estimates from NorStOP. These non-NZ sourced data provided relatively high estimates of the people in pain not due to knee OA, compared with unpublished 2013 NZHS micro-data,[15] and thus provide a conservative estimate of HRQoL decrement attributable to OA. The weighted HRQoL values for those in pain not due to OA, using three different distributions of moderate and severe pain, are shown in Supplementary Table A.

Supplementary Table A. HRQoL values for subjects in pain not due to OA.*

	**Age**	**Proportion in Pain[14]**	**Proportion in OA Pain**	**Proportion in Pain Not Due to OA**	**HRQoL Values**
Male	**40-44**	0.663	0.009	0.654	0.959
	**45-49**	0.663	0.038	0.625	0.728
	**50-54**	0.663	0.038	0.625	0.735
	**55-59**	0.663	0.060	0.603	0.727
	**60-64**	0.684	0.060	0.624	0.742
	**65-69**	0.684	0.116	0.568	0.725
	**70-74**	0.609	0.116	0.493	0.753
	**75-79**	0.609	0.131	0.479	0.772
	**80+**	0.574	0.131	0.444	0.745
Female	**40-44**	0.692	0.007	0.685	0.959
	**45-49**	0.692	0.037	0.655	0.717
	**50-54**	0.692	0.037	0.655	0.725
	**55-59**	0.692	0.092	0.600	0.717
	**60-64**	0.690	0.092	0.598	0.744
	**65-69**	0.690	0.167	0.523	0.735
	**70-74**	0.643	0.167	0.476	0.770
	**75-79**	0.643	0.214	0.429	0.778
	**80+**	0.656	0.214	0.442	0.763

*These estimates assume 90% moderate pain and 10% severe pain for those in pain not due to OA

E. Torrance transformation of HRQoL values

The HRQoL values in the NZ EQ-5D value set are based on health-state preferences elicited using the visual analogue scale (VAS) method; however, there have been reports suggesting that VAS scores may be downward biased relative to scores elicited using other methods such as the standard gamble (SG) sometimes used in international studies.[16] In order to allow comparison of the model output with other reports that utilized HRQoL values elicited using SG methodologies, (e.g. Losina et al.[17]), the VAS scores were transformed to approximate SG scores using a power transformation described by Torrance et al,[16] and transformed HRQoL values were derived. The power transformation described by Torrance et al.[16] used a power of 2.2 for the transformation. After transforming the values, we smoothed them within each pain group using linear regression. We used the same thresholds for each pain level as were used in the unadjusted analysis (i.e. less than 1 for no pain, 1 to 70 for moderate pain, and greater than 70 for severe pain). The Torrance-transformed HRQoL values for subjects with knee OA are shown in Supplementary Table B.

Supplementary Table B. Torrance-transformed HRQoL values for subjects in knee OA pain

	**Pain level**		
**Age**	**No pain or discomfort**	**Moderate pain or discomfort**	**Extreme pain or discomfort**
**40-44**	1.000	0.907	0.458
**45-49**	1.000	0.902	0.451
**50-54**	0.999	0.898	0.444
**55-59**	0.999	0.894	0.437
**60-64**	0.998	0.890	0.430
**65-69**	0.997	0.886	0.424
**70-74**	0.997	0.882	0.417
**75-79**	0.996	0.878	0.410
**80-84**	0.995	0.874	0.403

We applied both the untransformed and the transformed HRQoL values, and reported the latter as a more conservative estimate of the HRQoL values associated with knee OA pain.

We repeated the model runs to obtain race- sex- and age- specific per-person and population-wide QALY losses due to knee OA. The full results of the sensitive analysis using the Torrance-transformed HRQoL values are shown in Supplementary Figure A.

Supplementary Figure A. Ethnicity, sex, and age-specific per-person and population-based QALY losses due to OA, assuming 90% moderate pain for subjects in pain not due to OA, using Torrance-transformed HRQoL values

Race	Sex	Age	Proportion of persons with knee OA	QALE in persons with no knee OA	QALE in persons with knee OA	Per-person QALY losses in persons with OA	Population-based QALE losses in persons with knee OA
Non-Maori	Male	40-44	0.01	35.38	31.84	3.54	5,762
		45-49	0.05	31.11	28.21	2.91	18,537
		50-54	0.05	26.93	24.57	2.36	13,224
		55-59	0.06	22.90	21.02	1.88	12,976
		60-64	0.06	19.08	17.57	1.50	8,070
		65-69	0.11	15.46	14.28	1.18	9,432
		70-74	0.11	12.15	11.26	0.89	5,482
		75-79	0.13	9.21	8.57	0.64	3,722
		80-84	0.13	6.73	6.28	0.46	1,729
		**Total**		19.03	17.44	1.59	78,934
	Female	40-44	0.01	38.41	34.25	4.17	4,815
		45-49	0.04	34.11	30.60	3.51	21,512
		50-54	0.04	29.88	26.98	2.91	15,564
		55-59	0.10	25.76	23.36	2.40	28,064
		60-64	0.10	21.73	19.81	1.92	17,465
		65-69	0.17	17.87	16.36	1.50	18,897
		70-74	0.17	14.21	13.05	1.16	11,593
		75-79	0.22	10.84	10.02	0.82	9,493
		80-84	0.22	7.88	7.34	0.55	5,042
		**Total**		19.25	17.52	1.73	132,445
Maori	Male	40-44	0.01	28.76	26.31	2.45	405
		45-49	0.03	24.83	22.85	1.98	952
		50-54	0.03	21.09	19.52	1.57	583
		55-59	0.10	17.64	16.40	1.25	1,221
		60-64	0.10	14.57	13.55	1.01	666
		65-69	0.14	11.85	11.03	0.82	635
		70-74	0.14	9.41	8.77	0.64	308
		75-79	0.21	7.20	6.74	0.47	175
		80-84	0.21	5.40	5.05	0.35	57
		**Total**		15.44	14.32	1.12	5,002
	Female	40-44	0.01	32.05	29.04	3.00	826
		45-49	0.05	27.94	25.46	2.48	2,229
		50-54	0.05	23.99	21.96	2.03	1,368
		55-59	0.07	20.27	18.64	1.63	1,237
		60-64	0.07	16.90	15.57	1.32	679
		65-69	0.13	13.89	12.80	1.08	842
		70-74	0.13	11.17	10.32	0.85	421
		75-79	0.18	8.69	8.05	0.64	279
		80-84	0.18	6.52	6.08	0.44	100
		**Total**		18.94	17.36	1.58	7,982
**Totals**				19.03	17.38	1.65	224,364

Part II: Sensitivity Analysis Results

In the primary analysis, we assumed that 90% of people in pain not due to knee OA would be in moderate pain, and the remaining 10% would be in severe pain. We conducted a probabilistic sensitivity analysis by varying the proportion in moderate pain from 80% to 100% along a uniform distribution and performing 50 runs for each sex and race cohort. Using these 50 runs, we calculated the mean QALE for each cohort, as well as the 95% confidence interval for the QALY loss per person. The results of this analysis with the 95% uncertainty intervals are displayed in Supplementary Table C.

Supplementary Table C. QALY loss per person by sex and ethnicity from PSA varying the proportion of those in pain not due to knee OA who are in moderate pain from 80% to 100%

	**Weighted QALE in people with no knee OA**	**Weighted QALE in people with knee OA**	**Weighted QALE if people with OA didn't have knee OA**	**QALY loss per person with knee OA**	**95% CI for QALY loss per person**
**Non-Maori Male**	24.13	17.44	19.00	1.56	1.34 – 1.77
**Non-Maori Female**	26.83	17.52	19.25	1.73	1.51 – 1.95
**Maori Male**	21.46	14.32	15.41	1.10	0.91 – 1.28
**Maori Female**	24.15	17.36	18.95	1.59	1.34 – 1.85
**Total Population**	25.25	17.38	19.02	1.64	1.42 – 1.86

An additional sensitivity analysis performed using the prevalence estimates reported by the New Zealand Burden of Disease study are based on self-reported presence of physician-diagnosed OA without adjusting for self-report bias[9] as described in Section B, above, and in the main manuscript text. The full results from this sensitivity analysis, broken down by sex and race, and with 95% confidence intervals from the PSA analysis described above, are shown in Supplementary Table D.

Supplementary Table D. QALY loss per person by sex and ethnicity from PSA varying the proportion of those in pain not due to knee OA who are in moderate pain from 80% to 100%, based on unadjusted knee OA prevalence.

	Weighted QALE in people with no OA	Weighted QALE in people with OA	Weighted QALE if people with OA didn't have OA	QALY loss per person with OA	95% CI for QALY loss per person
**Non-Maori Male**	24.29	17.44	19.07	1.63	1.41 – 1.85
**Non-Maori Female**	27.13	17.52	19.35	1.83	1.63 – 2.04
**Maori Male**	21.61	14.32	15.47	1.15	0.96 – 1.34
**Maori Female**	24.31	17.36	19.03	1.67	1.43 – 1.91
**Total Population**	25.47	17.38	19.11	1.73	1.52 – 1.94

References

1. Statistics New Zealand. New Zealand Census of Population and Dwellings 2006 http://www.stats.govt.nz/Census/2006CensusHomePage.aspx: New Zealand Government; 2008 [cited 2016 18 May]. Available from: http://www.stats.govt.nz/Census/2006CensusHomePage.aspx.

2. Statistics New Zealand. New Zealand Life Tables: 2005-07 Wellington: Statistics New Zealand; 2009 [cited 2014 30 October, 2014]. Available from: http://www.stats.govt.nz/browse_for_stats/health/life_expectancy/new-zealand-life-tables-2005-07.aspx.

3. New Zealand Ministry of Health. A Portrait of Health: Online data tables of 2006/07 New Zealand Health Survey results http://www.health.govt.nz/publication/portrait-health-online-data-tables-2006-07-new-zealand-health-survey-results: New Zealand Government; 2008 [cited 2016 18 May]. Available from: http://www.health.govt.nz/publication/portrait-health-online-data-tables-2006-07-new-zealand-health-survey-results.

4. New Zealand Ministry of Health. Health Loss in New Zealand: A report from the New Zealand Burden of Diseases, Injuries and Risk Factors Study, 2006-2016. Wellington, New Zealand: Ministry of Health; 2013. 96 p.

5. New Zealand Burden of Diseases Statistical Annexe [Internet]. Ministry of Health. 2013. Available from: http://www.health.govt.nz/publication/new-zealand-burden-diseases-statistical-annexe.

6. United States National Health Interview Survey. United States National Health Interview Survey 2012 [cited 2015]. Available from: http://www.cdc.gov/nchs/nhis/about_nhis.htm.

7. Cross M, Smith E, Hoy D, Nolte S, Ackerman I, Fransen M, et al. The global burden of hip and knee osteoarthritis: estimates from the Global Burden of Disease 2010 study. Ann Rheum Dis. 2014;73(7):1323-30. doi: 10.1136/annrheumdis-2013-204763. PubMed PMID: 24553908.

8. Murray CJ, Vos T, Lozano R, Naghavi M, Flaxman AD, Michaud C, et al. Disability-adjusted life years (DALYs) for 291 diseases and injuries in 21 regions, 1990-2010: a systematic analysis for the Global Burden of Disease Study 2010. Lancet. 2012;380(9859):2197-223. doi: 10.1016/S0140-6736(12)61689-4. PubMed PMID: 23245608.

9. March LM, Schwarz JM, Carfrae BH, Bagge E. Clinical validation of self-reported osteoarthritis. Osteoarthritis Cartilage. 1998;6(2):87-93. doi: 10.1053/joca.1997.0098. PubMed PMID: 9692063.

10. PHARMAC. Prescription for Pharmacoeconomic Analysis: Methods for cost-utility analysis. Wellington: PHARMAC Pharmaceutical Management Agency, 2012 May 2012. Report No.

11. Devlin NJ, Hansen P, Kind P, Williams A. Logical inconsistencies in survey respondents' health state valuations -- a methodological challenge for estimating social tariffs. Health Econ. 2003;12(7):529-44. doi: 10.1002/hec.741. PubMed PMID: 12825206.

12. Devlin NJ, Hansen P, Kind P, Williams A. The health state preferences and logical inconsistencies of New Zealanders: A tale of two tariffs. CHE Discussion Paper 180. 2000;(July).

13. Devlin N, Hansen P, Herbison P. Variations in self-reported health status: results from a New Zealand survey. N Z Med J. 2000;113(1123):517-20. PubMed PMID: 11198514.

14. Thomas E, Peat G, Harris L, Wilkie R, Croft PR. The prevalence of pain and pain interference in a general population of older adults: cross-sectional findings from the North Staffordshire Osteoarthritis Project (NorStOP). Pain. 2004;110(1-2):361-8. doi: 10.1016/j.pain.2004.04.017. PubMed PMID: 15275787.

15. New Zealand Ministry of Health. New Zealand Health Survey 2012/2013 microdata Wellington, New Zealand: New Zealand Government; 2014 [cited 2016]. Available from: http://www.health.govt.nz/nz-health-statistics/national-collections-and-surveys/surveys/current-recent-surveys/new-zealand-health-survey - micro.

16. Torrance GW, Feeny D, Furlong W. Visual analog scales: do they have a role in the measurement of preferences for health states? Med Decis Making. 2001;21(4):329-34. PubMed PMID: 11475389.

17. Losina E, Walensky RP, Reichmann WM, Holt HL, Gerlovin H, Solomon DH, et al. Impact of obesity and knee osteoarthritis on morbidity and mortality in older Americans. Annals of Internal Medicine. 2011;154(4):217-26. Epub 2011/02/16. doi: 10.1059/0003-4819-154-4-201102150-00001. PubMed PMID: 21320937.
